# The Quality of Eggs from Rosa 1 Hens Fed Diets Containing Seeds of Legume Plants (*Lupinus luteus* L., *Lupinus angustifolius*, and *Pisum sativum*) in Two Laying Phases

**DOI:** 10.3390/ani10111942

**Published:** 2020-10-22

**Authors:** Joanna Kuźniacka, Jakub Biesek, Mirosław Banaszak, Małgorzata Grabowicz, Marek Adamski

**Affiliations:** 1 Department of Animal Breeding, Faculty of Animal Breeding and Biology, UTP—University of Science and Technology in Bydgoszcz, Mazowiecka 28, 85-084 Bydgoszcz, Poland; kuzniacka@utp.edu.pl (J.K.); miroslaw.banaszak@utp.edu.pl (M.B.); adamski@utp.edu.pl (M.A.); 2 Department of Physiology, Zoophysiotherapy and Animal Nutrition, Faculty of Animal Breeding and Biology, UTP-University of Science and Technology in Bydgoszcz, Mazowiecka 28, 85-084 Bydgoszcz, Poland; malgorzata.grabowicz@utp.edu.pl

**Keywords:** laying hens, laying phase, egg quality, lupins, pea

## Abstract

**Simple Summary:**

The use of legumes in diets for egg-laying hens exists as an alternative high-protein material in place of soybean meal, which will allow for wider production choices, including the elimination of genetically modified organism (GMO) products. The lack of negative impacts with legume usage on most of the examined traits and the improvement of the color of the yolk presents an attractive solution for the consumer market. The reduced content of anti-nutritional compounds in new varieties of lupins and peas is also a fact of the progress in agricultural production, including egg quality, which the producer can influence by feeding hens differently. The presented research indicates the possibility of using alternative feeding of laying hens in terms of the quality of table eggs.

**Abstract:**

This study analyzes the eggs’ quality from hens fed with alternative protein to soybean meal (SBM) in relation to laying phase. Here, 226 hens are divided into the control (I) and experimental (II) groups and reared for 50 weeks. There were two feeding phases (weeks 1–36; 37–50). Quality was assessed for 20 eggs from each group. The eggs are analyzed for their external and internal traits. A higher albumen weight in II and higher weights of the components were found in the 37–50 weeks for both groups, also in the percentage of yolk and albumen, however lower eggshell percentage was found for both groups, at the same time they were less durable and found to be thinner. In II, the La Roche color and the L*, b* were lower, but the a* increased, as did the albumen height. In the second phase, the La Roche and DSM color were higher, while the Haugh unit and albumen height decreased. Factors’ interaction was demonstrated for color. Legumes can partly substitute SBM in feed for hens. There were no negative effects on the eggs’ quality for most traits. The differences between the laying phases are due to the natural course and the physiology of laying hens.

## 1. Introduction

Soybean meal (SBM) is a popular protein-rich component of feed used in the diets of poultry and other monogastric animals. SBM is sourced from genetically modified plants, which poses a problem for the production of feed and the secure supply of protein because of the increasingly stronger criticism regarding genetically modified organisms (GMOs) and the forthcoming ban on the use of such raw materials in animal production. Some countries, like Poland, have good potential for growing protein-rich plants, such as legumes, including yellow lupins, narrow-leaved lupins, and peas. The dietary inclusion of these plants has historically been low because of their high contents of alkaloids and other antinutrients. Alkaloids reduce performance parameters, feed intake, and feed digestibility [1,2]; however, new cultivars of lupins and peas are characterized by significantly lower contents of alkaloids and high levels of protein. These protein-rich plants can be used in alternative poultry diets free from GMOs or with significantly reduced inclusion of GMOs. Peas are characterized by a 20–22% content of protein, with a beneficial amino acid composition and high energy value, and their dietary inclusion level can reach 15%. Sweet lupins (e.g., yellow and narrow-leaved) are rich in protein (43 and 32%, respectively). Their suggested proportion in feed mixtures is 10% [3,4]. Lupins are also characterized by a high content of carotenoids, which can improve the color of egg yolks. Yolk color is an important parameter for consumers. An intense yolk color is preferred by consumers, and pigments from lupins have good stability [4,5]. The aim of this study is to analyze the quality of eggs from hens kept in a semi-intensive system that are fed diets based on alternative sources of protein, as a substitute for soybean meal, in relation to the laying phase.

## 2. Materials and Methods 

The experiment was of an implementation nature, i.e., hens were kept on a small-scale family farm. Eggs were taken from hens that were kept on a production farm. This type of research does not require the consent of the ethics committee. Birds were not interfered with. The subject of the study was the quality of the raw material, i.e., consumption eggs from hens kept on the farm. Research on the quality of raw materials of animal origin does not legally require the permission of the ethics committee (directive No. 2010/63/EU).

### 2.1. Legume Seeds

Yellow lupin (*Lupinus luteus var.* Mister), narrow-leaved lupin (*Lupinus angustifolius var.* Sonet), and pea seeds (*Pisum sativum var.* Tarachalska) were obtained from plant breeding stations (Wiatrowo, Poland). The seeds were ground using a hammer mill (RG11 model, Zuptor, Gostyń, Poland) with a screen size of 2.0 mm. Table 1 presents the chemical compositions of the legumes that were used in this experiment.

### 2.2. Animals and Diets

For the trial, 18-week-old commercial Rosa 1 hens intended for semi-intensive egg production were used. The hens were assigned to two groups, each with one hundred and thirteen hens. No group replication was used due to the nature of the study under small-scale production conditions. The laying hens were kept on litter with a stocking density of 6 birds per 1 m^2^ with extra light in the henhouse of 4 W per 1 m^2^. The lighting program was set to 15.5 h of light per day. The hens were kept with paddock access. The birds received feed and water ad libitum. The different feeds were formulated with different levels of components per dietary ration for the two periods of egg production (weeks 1–36, weeks 37–50) (Table 2). The control group (I) received a balanced feed containing 41.7% soybean meal (GMO). Birds from the experimental group (II) were fed on a balanced mixture containing vegetable protein in a ground form sourced from peas (Tarchalska, 11.10%), yellow lupins (Mister, 24.90%), and narrow-leaved lupins (Sonet, 22.20%). Crude protein contents and the metabolizable energy (ME) values for the control and experimental concentrates are presented in Table 3. The feed rations were established according to the recommendations of Smulikowska and Rutkowski [6]. The crude protein and ME content in diets are presented in Table 2. Additionally, in Table 4, the antinutrient compounds in legumes are presented. All the feed elements (Table 1, Table 2, Table 3 and Table 4) were provided by the feed manufacturer that produced the feed in the presented studies. In the presented studies, the chemical composition of feed was not analyzed by the authors, while the methods used to provide the data from the Feed Manufacturing Plant presented in the Tables mentioned above are consistent with the methods described by Kuźniacka et al. [7].

### 2.3. Laying Performance

The performance of the laying hens was monitored. Birds were weighed at the beginning and at the end of the experiment, and feed intake was also measured. The number of eggs was measured during the whole laying period. After laying, number of eggs per hen, as well as the daily feed intake (g/d) and daily feed intake per hen (g) were calculated. Thanks to these results, the mean feed intake for one egg (g) could be found.

### 2.4. Egg Quality 

Eggs were collected daily. In total, 20 eggs from each group were analyzed for the egg quality parameters every 4 weeks for 50 weeks. Eggs were weighed on a RADWAG PS 1000.R2 scale with accuracy to the nearest 0.001 g, and the heights and widths of eggs were measured with a Metr-ISO electronic caliper. Egg shape index values were calculated using the formula (width/height) × 100. Egg shell hardness was tested with an egg force reader produced Orka Food Technology Ltd. [N]. An EggAnalyzer from Orka Food Technology Ltd. was used to analyze the egg components and quality, i.e., the height of albumen, score in Haugh units, and yolk color. Yolk color was assessed using a Konica Minolta colorimeter, model CR400, Japan. The device was calibrated using the white calibration plate No. 21033065 and a D_65_ Y_86.1_ x_0.3188_ y_0.3362_ scale. Color was graded according to the CIE L*a*b* system (L* = lightness, a* = redness, and b* = yellowness) [8] and a 15-tone DSM Yolk Color Fan. Yolks were weighed on a RADWAG PS 1000.R2 scale with accuracy to the nearest 0.001 g. The egg morphologies and components (the proportion of yolk, albumen, and shell) were analyzed. Shells were dried in a SUP-100 M oven at 105 °C for 3 h and then weighed and measured for thickness with a TSS electronic micrometer. 

### 2.5. Statistical Evaluation

Numerical data were processed with statistical software (STATISTICA 10.0 pl, ver. 2011) (StatSoft, Kraków, Poland) [9]. Mean values (x) for each treatment group and laying phase and total standard error of the mean (SEM) values were calculated with subclass effects. The statistics were analyzed with a two-way analysis of variance (ANOVA) and the significance of difference values were verified with a post-hoc Tukey’s test at the significance level of *p* < 0.05 for the treatment group and laying phase. *P*-values less than 0.05 were statistically significantly different. The diet–laying phase interaction was calculated by a one-dimensional effect for each variable at *p* < 0.05. Each individual egg was the basic experimental unit used to obtain mean values in groups and to perform further stages of analysis. Laying performance was not calculated due to the statistical post-hoc test, and only mean values were obtained.

## 3. Results

### 3.1. Laying Performance

The mortality of hens was not more than 1% in the whole flock. In Table 5, the average productivity parameters values, including the average number of eggs per hen, the hen’s feed consumption, the hen’s daily feed consumption and the feed consumption per egg production. These data were not statistically analyzed. In both groups, similar mean values were observed.

### 3.2. Eggs Quality

In the experimental group (II), a significantly higher weight of albumen eggs was shown than in the control group (I) (*p* = 0.018), while for the other characteristics, no significant differences were found between the diet groups for the other characteristics (*p* > 0.05) (Table 6).

On the other hand, between the weeks of laying, greater weights of the eggs, yolks, albumen, and eggshells were shown, as well as greater percentages of the yolk and egg albumen in the second stage of laying (36–50 weeks) (*p* < 0.05), while the percentage of the eggshell was higher in the first stage of laying (1–36 weeks, *p* < 0.001). Only the egg shape index was similar throughout the laying period (*p* = 0.094).

Analyzing the results of the strength of the eggshell and its thickness (Table 7), significantly higher strength values and a thicker eggshell in the first laying period (*p* < 0.001) were shown. There was no mutual relationship (interaction) between the feeding and laying phases on the morphological features of the egg and the parameters of the eggshell (*p >* 0.05).

Table 8 shows the yolk color results and the heights of the thick albumen and Haugh units of the tested eggs. In terms of the different nutrition scenarios, it was shown that eggs from hens fed a diet of legume seeds had a significantly higher color value measured with the La Roche scale (*p* < 0.001). However, when analyzing the color in the CIE Lab system, in group II, lower values of the L* and a* (redness) parameters were found compared to group I, as well as a significantly higher saturation of yellow (b*) (*p* < 0.05). Group II eggs were also characterized by a significantly lower height of thick albumen (*p* < 0.001).

In the second laying period (36–60 weeks), a significantly higher color value on the La Roche scale was found, as well as for the DSM scale (*p* < 0.05). In the same period, a significantly lower height of thick albumen and a lower Haugh value (*p* < 0.05) were demonstrated. 

A statistically significant interaction between the diet and laying phase was demonstrated in the color parameters measured on the La Roche scale (*p* < 0.001), DSM YolkFan scale (*p* = 0.004), and the parameters of CIE Lab system, including L* (*p* = 0.016), a* (*p* = 0.007), and b* (*p* = 0.012). No statistically significant differences or interactions between the factors were found in the remaining features (*p >* 0.05).

## 4. Discussion

The effects of the substitution of soybean meal with legume seeds [10,11,12,13,14,15,16] or other protein sources, such as mulberry leaves [17] or insect meal [18,19], in poultry diet on the quality of poultry products has been investigated by many researchers.

Kaczmarek et al. [20] used soybean meal, blue lupins (narrow-leaved), and yellow lupins in the diets of broiler chickens. The analysis results for the chemical compositions of seeds showed that blue lupins had a similar content of crude protein (CP) to those found in the two groups in our study (30.00 and 30.30%, respectively), as well as the acid detergent fiber (ADF) and neutral detergent fiber (NDF) contents in both lupin cultivars. However, our result for the CP content in yellow lupins was 4.67% higher than in the cited research. The total alkaloid content was lower in our study (YL, yellow lupin = 0.00085 g/kg; NLL, narrow-leafed lupin = 0.0017 g/kg), whereas the cited study shows different results (YL = 0.22 g/kg and NLL = 0.20 g/kg). This may be due to the progress in breeding for new lupin varieties with reduced alkaloid content [21]. The use of narrow-leaved lupins (*var.* Boruta), yellow lupins (*var.* Mister), and peas (*var.* Muza) in egg-laying hen production has been presented by Rutkowski et al. [11]. They used other varieties of narrow-leaved lupins and peas characterized by higher crude protein contents (36.88 and 27.57%, respectively) than those used in this study (30.30 and 20.38%, respectively). The aim of the study of Księżak et al. [22] was the evaluation of the concentration of nutrients in the seeds of few faba bean and pea varieties. They compared the pea *var.* Boruta and *var.* Tarchalska. Both varieties had similar contents of crude protein (21.70 and 21.50%, respectively). The total alkaloid content in yellow lupins is lower than in narrow-leaved lupins [11,23], and this was confirmed in our research. The content of antinutritive substances may negatively affect some productivity parameters and egg quality traits [11,15]. Similarly, McNeil et al. [24] reported that the use of pea seeds in broiler chicken diets may influence growth performance. The use of legumes in poultry diet has been the subject of studies from many years ago. Additionally, Castano and Perez-Lanzac [25] observed that there is a negative relationship between legumes and the productivity parameters of laying hens. However, over a period of approximately 30 years, legume varieties have changed to feature a significantly lower content of anti-nutritional ingredients, and this is not a problem in poultry nutrition.

In the study about the use of yellow lupin seeds for laying hens presented by Zduńczyk et al. [26], they showed that the dietary inclusion of yellow lupin at 30% level in feed had no negative effect on bird performance. Rutkowski et al. [11] analyzed eggs from Hy-Line Brown hens fed three different diets. Egg weight for the control hens fed on a mixture containing 18.02% of soybean meal was significantly higher (57.92 g) than in experimental groups, whose diets contained 19.48% (group II, 55.94 g) and 27.68% (group III, 54.99 g) legume seeds. The present study did not reveal such differences. The aforementioned study concluded that the use of 27.68% legume seeds (pea *var.* Boruta, yellow lupin *var.* Mister, narrow-leaved lupin *var.* Muza) in the diet of laying hens has a negative influence on performance results. However, a lower content of these seeds (19.48%) could be accepted to substitute soybean meal. Lee et al. [12] reported no significant differences in the weights of eggs produced by hens fed a diet containing soybean meal, whole lupin seeds, and dehulled lupin seeds. This is consistent with findings the findings of Krawczyk et al. [13]. Rutkowski et al. [11] reported an increased the Haugh unit score and a stronger pigmentation of yolk, but significantly thinner shells in eggs from hens fed a balanced diet with the inclusion of lupin seeds (27.68%). Krawczyk et al. [13] found no significant differences in the shell thickness and strength. Similarly, no significant differences were found in our study. Shell thickness significantly decreased in the second feeding phase within the two groups here. In conclusion, the level of dietary lupin has to be carefully graded, because excess addition presents a negative effect on egg quality. In our study, egg yolk color (yellowness) differed significantly between groups, while Lee et al. [16] reported no differences. The redness of yolk in the group fed lupin seeds was significantly higher than in the control group. Our study also revealed intergroup differences. A stronger pigmentation of egg yolk in the experimental group was also reported by Laudadio and Tufarelli [14], as well as Lokaewmanne et al. [17], who investigated the effects of a diet containing alfalfa and yellow corn grain. A stronger pigmentation of yolk (increased redness) may also result from a higher concentration of carotene in seeds [27,28]. Cuiurescu and Pană [29] analyzed the effect of a diet containing field peas as an alternative to soybean meal and enzyme supplementation on the performance of laying hens. In their study, different dietary treatments had no significant effect on the albumen height, yolk weight, or egg shell weight, except for yolk color, which was more strongly saturated in hens fed the pea diet. Fru-Nji et al. [30] reported similar findings for diets containing peas.

In addition to studies on the impact of nutrition with legumes, the impact of the laying hen age on egg quality has also been studied. The research of Drażbo et al. [31] focused on Lohmann Brown hens fed with narrow-leafed lupin at 10 and 20%. Egg weight was not found to be dependent on the age of the hens during the laying period (4–20 weeks). Similarly, Park et al. [32] did not show a statistically significant effect of the laying period and diet on the egg weight of Hy-Line Brown hens. However, the relationship between egg weight and laying weeks (feeding phases) has been demonstrated in our own study with Rosa 1 laying hens. Similar results could be found with other genotypes of hens [33]. The differences between the laying phases in egg characteristics could be related to the natural laying course and to the physiology of the laying hens. Changes in the intensity of the yolk color are related to xanthophylls and lutein in the feed, as well as the lipids content of the yolk. As the production cycle passes, the lipids-soluble carotenoid pigments are absorbed more in the intestine of laying hens [34].

## 5. Conclusions

In this study, the effect of soybean meal (SBM) substitutes in the diets of laying hens in terms of laying performance and egg quality has been analyzed. It has been shown that protein sources other than SBM at the level of approximately 26.19% in the first phase of feeding and approximately 23.28% in the second phase of feeding can be included in diet of the hens considered here. For both feeding groups, the production results of laying hens were similar, which is important from the producer’s point of view. Importantly, in the consumer market, nutrition based on legume seeds does not have a negative impact on the weight of the egg and the Haugh unit value, indicating that eggs from both groups are characterized by a similar level of freshness and suitability for consumption. The higher yellowness in the test group eggs may be a positive feature, since consumers are driven by the choice of products, inter alia, by their eyesight, and this may be a feature that encourages purchasing eggs with an intense yellow yolk. The colors of the yolks also depended on the age of the hens during laying, and it was concluded here that more yellow egg yolks were produced in the later laying stages. The proposed balanced ration for legume seeds as an alternative to soybean meal should be recommended as a partial replacement.

## Figures and Tables

**Table 1:** Chemical compositions of legume seeds (% of dry matter) ^1^.

Components	Pea (Tarchalska)	Yellow Lupin (Mister)	Narrow-Leaved Lupin (Sonet)
Crude protein	20.38	42.47	30.30
ADF ^2^	9.09	19.39	20.46
NDF ^3^	16.50	25.16	25.5
Crude fat	1.03	3.52	4.87
Starch	59.01	−	−
Amino acids, % protein			
Asp. Ac.	12.23	11.21	9.98
Thr	3.50	3.66	3.41
Ser	4.53	5.51	4.94
Glut. Ac.	15.77	25.22	19.54
Pro	3.80	3.61	3.71
Cys	0.86	1.91	0.82
Met	0.57	0.53	0.36
Gly	4.11	4.28	4.05
Ala	4.11	3.54	3.37
Val	4.85	4.04	3.92
Iso	3.94	4.3	4.01
Leu	7.06	8.85	6.89
Tyr	2.83	3.09	3.24
Phe	4.59	4.42	4.02
His	2.73	3.05	2.69
Lys	7.91	6.66	5.45
Arg	12.23	15.11	11.64

^1^ Each value represents mean of two replicates. ^2^ ADF—acid detergent fiber. ^3^ NDF—neutral detergent fiber.

**Table 2:** Levels of feed components in hen diets and throughout the whole egg production period (50 weeks).

Components	Group 1	
	I	II
Weeks 1–36		
Wheat (%)	55	55
Concentrate (%)	45	45
Weeks 37–50		
Wheat (%)	60	60
Concentrate (%)	40	40
ME (mJ/kg of feed)	11.30–11.60	
CP (%)	15.00–17.00	

CP: Crude protein; ME: Metabolizable energy.

**Table 3:** Component contents in concentrates (%).

Components	Groups ^1^	
	I	II
Soybean meal	41.70	−
Pea (Tarchalska)	−	11.10
Yellow lupin (Mister)	−	24.90
Narrow-leaved lupin (Sonet)	−	22.20
Maize	21.30	4.44
Fodder chalk	18.68	18.90
Rapeseed oil	13.30	12.20
Monocalcium phosphate	2.25	3.10
NaHCO_3_	0.52	0.78
DL-methionine	0.40	0.47
NaCl	0.44	0.29
L-lysine	0.56	0.56
Threonine	0.42	0.43
Tryptophan	0.10	0.09
L-valine	0.33	0.49
ME (mJ/kg of feed)	10.80	10.20
CP (%)	20.00	22.10

^1^ I—control group fed with soybean meal; II—experimental group fed with legume seeds. Feed rations were established based on recommendations by Smulikowska and Rutkowski [6].

**Table 4:** Antinutrients in legume seeds used in feed for laying hens.

Antinutrients	Pea (Tarchalska)	Yellow Lupin (Mister)	Narrow-Leaved Lupin (Sonet)
Antinutrients	−	−	−
Total alkaloids, g/kg	−	0.00085	0.0017
Oligosaccharides, g/kg	62.91	134.27	58.06
Rafinose, g/kg	8.36	10.21	7.07
Stachyose, g/kg	27.13	82.80	37.23
Verbascose, g/kg	27.42	41.26	13.76
Phosphorus phytic, (%)	0.24	0.74	0.48
Tannin, g/kg	0.021	−	−

**Table 5:** Mean values of the productivity parameters of laying hens during the experiment *.

Feeding Phase	Weeks 1–36		Weeks 37–50	
Group ^1^	I	II	I	II
Number of eggs per hen	191.00	192.00	126.00	130.00
Feed intake per hen (kg)	20.50	20.90	11.40	11.50
Daily feed intake per hen (g)	127.00	130.00	116.20	117.60
Feed intake per egg (g)	167.80	170.80	193.50	187.00

^1^ I—control group fed with soybean meal; II—experimental group fed with legume seeds; *, the data presented in Table 5 were not statistically analyzed, which is equivalent to no significant differences.

**Table 6:** Parameters of egg morphology and egg components.

Traits *n* = 20 Eggs Per Group		Groups				SEM	*p*-Value		
		I	II	Weeks			Diet	Laying Phase	Diet–Laying Phase
				1–36	37–50				
Egg weight (g)		63.17	63.93	59.55 ^b^	68.89 ^a^	0.32	0.130	<0.001	0.248
Egg shape index (%)		73.88	73.66	73.98	73.48	0.15	0.459	0.094	0.758
Weight and proportion in egg	Yolk (g)	17.86	17.73	15.80 ^b^	20.46 ^a^	0.15	0.550	<0.001	0.399
	Yolk (%)	28.07	27.61	26.41 ^b^	29.76 ^a^	0.15	0.071	<0.001	0.882
	Albumen (g)	39.82 ^b^	40.66 ^a^	38.38 ^b^	42.71 ^a^	0.20	0.018	<0.001	0.346
	Albumen (%)	63.20	63.68	64.57 ^a^	61.92 ^b^	0.14	0.066	<0.001	0.833
	Eggshell (g)	5.49	5.55	5.37 ^b^	5.72 ^a^	0.03	0.305	<0.001	0.209
	Eggshell (%)	8.73	8.71	9.02 ^a^	8.32 ^b^	0.03	0.713	<0.001	0.767

I—control group fed with soybean meal; II—experimental group fed with legume seeds; SEM—standard error of the mean; n—number of eggs per group analyzed; a,b—mean values marked in columns with different letters are significantly different within the diet group or laying phase (*p* < 0.05).

**Table 7:** Parameters of eggshells.

Traits *n* = 20 Eggs Per Group	Groups				SEM	*p*-Value		
	I	II	Weeks			Diet	Laying Phase	Diet–Laying Phase
			1–36	37–50				
Strength of the eggshell (N)	30.02	29.29	30.86 ^a^	28.05 ^b^	0.39	0.338	<0.001	0.925
Thickness of the eggshell (mm)	0.311	0.311	0.318 ^a^	0.301 ^b^	0.03	0.932	<0.001	0.449

I—control group fed with soybean meal; II—experimental group fed with legume seeds; SEM—standard error of the mean; n—number of eggs per group analyzed; a,b—mean values marked in columns with different letters are significantly different within the diet group or laying phase (*p* < 0.05).

**Table 8:** Parameters of egg components.

Traits *n* = 20 Eggs Per Group		Groups				SEM	*p*-Value		
		I	II	Weeks			Diet	Laying Phase	Diet–Laying Phase
				1–36	37–50				
La Roche		3.49 ^b^	4.09 ^a^	3.36 ^b^	4.36 ^a^	0.07	<0.001	<0.001	<0.001
DSM YolkFan scale		5.17	5.85	5.31 ^b^	5.77 ^a^	0.07	0.401	0.001	0.004
Color	L *	55.18 ^a^	54.32 ^b^	54.98	54.44	0.14	0.003	0.056	0.016
	a *	−4.04 ^a^	−3.60 ^b^	−3.93	−3.67	0.07	0.002	0.062	0.007
	b *	32.38 ^b^	33.99 ^a^	32.96	33.49	0.22	<0.001	0.221	0.012
Albumen height (mm)		5.17 ^a^	5.06 ^b^	5.40 ^a^	4.74 ^b^	0.07	<0.001	<0.001	0.582
Haugh units		63.60	61.64	64.64 ^a^	59.93 ^b^	0.76	0.191	0.001	0.107

I—control group fed with soybean meal; II—experimental group fed with legume seeds; ^2^ L*—lightness; a*—redness; b*—yellowness; SEM—standard error of the mean; n—number of eggs per group analyzed; a,b—mean values marked in columns with different letters are significantly different within the diet group or laying phase (*p* < 0.05).
